# From Inclusion Complexes to Innovative Therapies: Cyclodextrins and Their Pharmaceutical Applications

**DOI:** 10.3390/pharmaceutics18030303

**Published:** 2026-02-28

**Authors:** Diana-Maria Trasca, Denisa Floriana Vasilica Pirscoveanu, Renata-Maria Varut, Ion Dorin Pluta, George-Alin Stoica

**Affiliations:** 1 Department of Internal Medicine, University of Medicine and Pharmacy of Craiova, 200349 Craiova, Romania; diana.trasca@umfcv.ro; 2 Department of Neurology, Faculty of Medicine, University of Medicine and Pharmacy of Craiova, 200349 Craiova, Romania; denisa.pirscoveanu@umfcv.ro; 3 Research Methodology Department, Faculty of Pharmacy, University of Medicine and Pharmacy of Craiova, 200349 Craiova, Romania; 4 Faculty of Medical and Behavioral Sciences, Constantin Brâncuși University of Târgu Jiu, 210185 Târgu Jiu, Romania; 5 Department of Pediatric Surgery, Faculty of Medicine, University of Medicine and Pharmacy of Craiova, 200349 Craiova, Romania; alin.stoica@umfcv.ro

## 1. Introduction

Originally discovered over a century ago, cyclodextrins (CDs) have evolved from biochemical curiosities into indispensable enablers of modern drug delivery. In the past few decades, their pharmaceutical potential has expanded exponentially. CDs are cyclic oligosaccharides with a hydrophilic exterior and hydrophobic cavity; this unique structure allows them to form inclusion complexes with drug molecules, encapsulating hydrophobic compounds in their central cavity [1,2,3]. By doing so, CDs can dramatically increase a drug’s aqueous solubility, shield labile active ingredients from chemical or enzymatic degradation, and modulate the release profiles of drugs. They can even reduce drug volatility and mask unpleasant odors or tastes, improvements that translate into enhanced therapeutic efficacy, safety, and patient compliance [4,5].

Over time, cyclodextrin applications have transcended traditional formulation roles. Beyond simply solubilizing poorly water-soluble drugs, CDs are now being explored in advanced therapeutic contexts such as targeted cancer therapy, gene delivery, and treatments for neurodegenerative diseases [6,7,8,9,10]. Their proven biocompatibility and relatively low toxicity underlie several regulatory approvals of CD-containing products and have encouraged innovative uses in in vivo delivery systems. Chemical modifications of the native α-, β-, and γ-cyclodextrin molecules have further broadened their utility, yielding derivatives tailored for higher specificity, capacity, or biological functions. Indeed, with ongoing advances in CD synthesis and functionalization, these “molecular containers” continue to offer novel solutions to longstanding drug formulation challenges [10,11,12,13,14].

This Special Issue, “Cyclodextrins and Their Pharmaceutical Applications”, showcases the diversity of current research on pharmaceutical CDs. The eight contributions in this issue, a mix of original research articles and comprehensive reviews, underscore how CDs can enhance drug solubility, stability, and bioavailability, enable novel drug delivery systems, potentiate antimicrobial therapies, incorporate volatile bioactives such as essential oils, and maintain favorable safety profiles in complex formulations. In the following sections, we provide an overview of these contributions and discuss their implications for the future of cyclodextrin-based pharmaceutical science.

Recent advances in supramolecular chemistry, materials science, and pharmaceutical nanotechnology have further accelerated interest in cyclodextrin-based systems, positioning them at the interface between classical formulation science and next-generation therapeutic strategies [15,16,17].

## 2. Overview of Published Work

The articles collected in this Special Issue reflect both the maturity and the ongoing innovation of cyclodextrin research, spanning fundamental inclusion complexation, applied formulation science, and advanced drug delivery platforms.

Several contributions in this Special Issue focus on improving the solubility and stability of poorly water-soluble drug molecules through cyclodextrin inclusion strategies. These studies highlight the role of cyclodextrins as enabling excipients capable of enhancing physicochemical properties while maintaining formulation compatibility and biological performance.

Contribution (1) reports the design of an inclusion complex between sildenafil, a poorly water-soluble BCS class II drug, and hydroxypropyl-β-cyclodextrin (HPβCD). The authors confirmed the formation of a 1:1 host–guest complex and systematically evaluated its compatibility with commonly used pharmaceutical excipients. No chemical incompatibilities were observed under ambient conditions, supporting the suitability of this complex for further formulation development, while only minor interactions emerged under accelerated thermal stress. This study provides a solid preformulation framework demonstrating that cyclodextrin complexation can enhance solubility without compromising formulation stability.

Contribution (2) explores β-cyclodextrin inclusion complexes of N-(2-bromo-phenyl)-2-hydroxy-benzamide derivatives with the aim of improving antimicrobial and anti-inflammatory activity. Structural and thermal analyses confirmed successful complex formation, while biological evaluations showed enhanced antimicrobial effects, particularly against Gram-positive bacteria, along with improved anti-inflammatory responses compared to the free compounds. These findings indicate that cyclodextrin encapsulation can significantly enhance the biological performance of lipophilic therapeutic molecules.

Another important thematic cluster addresses the encapsulation of natural, volatile, or biologically sensitive compounds, with particular emphasis on antimicrobial activity and stability enhancement. Cyclodextrin-based systems are shown to mitigate volatility, protect labile constituents, and improve the pharmaceutical usability of natural bioactives.

The pharmaceutical potential of cyclodextrins for stabilizing volatile natural products is demonstrated in Contribution (3), which focuses on essential oils rich in bioactive constituents. The authors formulated eugenol, eucalyptol, and clove essential oil into β-cyclodextrin inclusion complexes using different preparation techniques. Cyclodextrin encapsulation reduced volatility, preserved the chemical integrity of key components, and resulted in improved antimicrobial performance. This work highlights cyclodextrins as valuable excipients for converting volatile, poorly soluble natural products into more stable and pharmaceutically viable formulations.

In Contribution (4), a γ-cyclodextrin-based metal–organic framework (γ-CD-MOF) is introduced as a supramolecular carrier for muscone, a natural compound with pharmacological relevance but limited clinical application due to poor solubility and moderate stability. Encapsulation within the biodegradable γ-CD-MOF enhanced muscone solubility and protected it against thermal and photodegradation. Importantly, the system exhibited favorable biocompatibility in cell-based evaluations, supporting the potential of cyclodextrin-derived frameworks as advanced drug delivery platforms.

Beyond classical inclusion complexation, several studies illustrate the role of cyclodextrins as functional building blocks in advanced drug delivery systems. These approaches emphasize the transition of cyclodextrins from passive solubilizers to active structural components enabling controlled release, enhanced bioavailability, and multifunctional performance. Cyclodextrin-based materials are further explored in Contribution (5), which presents a polymer-free electrospinning approach using a β-cyclodextrin–oligolactide conjugate to deliver magnolol and honokiol. These bioactive polyphenols are known for antioxidant and antibacterial properties but suffer from poor solubility and limited bioavailability. Incorporation into cyclodextrin-based nanofibrous mats resulted in amorphous, readily releasable formulations with prolonged antioxidant activity and enhanced antibacterial effects. This study demonstrates how cyclodextrin derivatives can function simultaneously as carriers and structural components in advanced delivery systems.

Complementing the experimental studies, three review articles provide broader perspectives on cyclodextrin-based pharmaceutical technologies, addressing both fundamental concepts and practical formulation considerations. Together, these contributions discuss emerging applications, translational challenges, and regulatory aspects relevant to the safe and effective use of cyclodextrins in modern drug products. Broader perspectives on cyclodextrin complexation are provided in Contribution (6), a comprehensive review focusing on cyclodextrin complexes with bioactive secondary metabolites. The review summarizes strategies for improving solubility, stability, and bioavailability of natural compounds through cyclodextrin inclusion and composite delivery systems. It also discusses pharmacokinetic and pharmacodynamic implications, along with current limitations and future research directions, emphasizing the role of cyclodextrins in bridging the gap between bioactivity and clinical applicability.

Contribution (7) offers a wide-ranging review of cyclodextrins in modern pharmaceutical applications, highlighting their impact on drug solubility, stability, bioavailability, and controlled release across various routes of administration. The authors also discuss emerging cyclodextrin-based systems, including nanoparticulate and targeted delivery approaches, underscoring the evolving role of cyclodextrins as multifunctional tools in contemporary therapeutics.

Finally, Contribution (8) addresses an important formulation and regulatory consideration: the interaction between cyclodextrins and antimicrobial preservatives in multidose pharmaceutical products. The review analyzes how cyclodextrin complexation may reduce preservative availability and antimicrobial efficacy and discusses formulation strategies to mitigate these effects. This contribution provides essential guidance for the safe and effective design of cyclodextrin-containing drug products.

Taken together, Contributions (1)–(8) illustrate the breadth and depth of current cyclodextrin research, encompassing fundamental inclusion complexation, advanced delivery systems, and practical formulation challenges. Collectively, these studies reinforce the versatility of cyclodextrins and their growing importance in the development of safe, effective, and innovative pharmaceutical therapies.

## 3. Future Perspectives

As illustrated by the contributions in this Special Issue, cyclodextrin-based technologies are poised to play an increasingly prominent role in pharmaceutical research and development. The Guest Editors have identified several key trends that are likely to shape the future of this field.

First, continued innovation in cyclodextrin-based delivery systems is expected. Emerging platforms such as cyclodextrin metal–organic frameworks, nanofibers, nanosponges, and nanoparticle systems suggest that cyclodextrins will increasingly function as structural and functional components of drug carriers, rather than solely as solubilizing excipients. These developments may enable more effective treatments for complex diseases and support the delivery of advanced therapeutics.

Second, safety evaluation and regulatory considerations will remain critical as cyclodextrin applications expand. Although many cyclodextrins are already approved for pharmaceutical use, new derivatives, higher doses, and alternative routes of administration will require careful toxicological assessment. A deeper understanding of cyclodextrin pharmacokinetics and formulation interactions will be essential to support broader clinical translation.

Third, cyclodextrin-based formulations align well with the growing emphasis on patient-centric and personalized medicine. By improving oral bioavailability, masking unpleasant sensory properties, and reducing irritation, cyclodextrins can enhance patient adherence and comfort. Their versatility also supports the development of tailored formulations for specific patient populations, including pediatric and geriatric patients.

In conclusion, the contributions collected in this Special Issue highlight the remarkable versatility and impact of cyclodextrins in pharmaceutical science—from improving the performance of individual drug molecules to enabling advanced therapeutic platforms. We hope this collection will stimulate further interdisciplinary research and innovation, supporting the continued evolution of cyclodextrins from classical inclusion complexes to integral components of next-generation pharmaceutical therapies.

We trust that this Special Issue will serve not only as a snapshot of current achievements, but also as a catalyst for future interdisciplinary collaborations advancing cyclodextrin-based pharmaceutical innovation.

## References

[1] Niţu E.T., Ridichie A., Temereancă C., Mitrofan I., Buliga L., Simu S., Muntean C., Rusu G., Ledeţi I., Ledeţi A. (2025). Study of Carvedilol–β-Cyclodextrin Derivatives Interactions. Processes.

[2] Zhang W., Zhou T.-P., Zou W., Wang Y., Wang K., Yang Y., Liu C., Tu Z., Liu Q., Yuan Y. (2025). Formation of β-Cyclodextrin Inclusion Complexes with a Series of Structurally Related Parabens: Preparation, Physicochemical Characterization and Antifungal Properties. Carbohydr. Polym. Technol. Appl..

[3] Rajamohan R., Muthuraja P., Murugavel K., Mani M.K., Prabakaran D.S., Seo J.H., Malik T., Lee Y.R. (2025). Significantly Improving the Solubility and Anti-Inflammatory Activity of Fenofibric Acid with Native and Methyl-Substituted Beta-Cyclodextrins via Complexation. Sci. Rep..

[4] Omidian H., Akhzarmehr A., Gill E.J. (2025). Cyclodextrin–Hydrogel Hybrids in Advanced Drug Delivery. Gels.

[5] Angelova S., Pereva S., Dudev T., Spassov T. (2025). Cyclodextrins’ Internal Cavity Hydration: Insights from Theory and Experiment. Inorganics.

[6] Liñán-Atero R., Aghababaei F., García S.R., Hasiri Z., Ziogkas D., Moreno A., Hadidi M. (2024). Clove Essential Oil: Chemical Profile, Biological Activities, Encapsulation Strategies, and Food Applications. Antioxidants.

[7] Musuc A.M. (2024). Cyclodextrins: Advances in Chemistry, Toxicology, and Multifaceted Applications. Molecules.

[8] Su Q., Su W., Xing S., Tan M. (2024). Enhanced stability of anthocyanins by cyclodextrin–metal organic frameworks: Encapsulation mechanism and application as protecting agent for grape preservation. Carbohydr. Polym..

[9] Zhai Y., Zhang D., Gao L. (2024). Magnolol-based sustainable, high-strength, shape-memory, transparency, and UV-shielding thermosetting polyurethane networks. Eur. Polym. J..

[10] Blaj D.-A., Balan-Porcarasu M., Harabagiu V., Peptu C. (2024). Synthesis of β-Cyclodextrin Derivatives Substituted at Larger or Smaller Rims via Amine-Catalyzed Ring-Opening Oligomerization of ε-Caprolactone. Carbohydr. Polym..

[11] Kfoury M., Lichtfouse E., Fourmentin S. (2025). The revival of cyclodextrins as active pharmaceutical ingredients. Environ. Chem. Lett..

[12] Nicolaescu O.E., Belu I., Mocanu A.G., Manda V.C., Rău G., Pîrvu A.S., Ionescu C., Ciulu-Costinescu F., Popescu M., Ciocîlteu M.V. (2025). Cyclodextrins: Enhancing Drug Delivery, Solubility and Bioavailability for Modern Therapeutics. Pharmaceutics.

[13] Pereira de Lima E., Laurindo L.F., Catharin V.C.S., Direito R., Tanaka M., Santos German I.J., Barbalho Lamas C., Landgraf Guiguer E., Cressoni Araújo A., Ragassi Fiorini A.M. (2025). Polyphenols, Alkaloids, and Terpenoids Against Neurodegeneration: Evaluating the Neuroprotective Effects of Phytocompounds Through a Comprehensive Review of the Current Evidence. Metabolites.

[14] Qin L., Tu J., Zhao J., Zhang Y., Li T., Zhang Y., Zhang P., Ling G., Ji J. (2025). Dual-targeted and esterase-responsive cyclodextrin-based host-guest nanocomposites for enhanced antitumor therapy. Colloids Surf. B Biointerfaces.

[15] Şuta L.-M., Ridichie A., Ledeţi A., Temereancă C., Ledeţi I., Muntean D., Rădulescu M., Văruţ R.-M., Watz C., Crăineanu F. (2024). Host–Guest Complexation of Itraconazole with Cyclodextrins for Bioavailability Enhancement. Pharmaceutics.

[16] Volkova T., Simonova O., Perlovich G. (2024). Controlling the Solubility, Release Rate and Permeation of Riluzole With Cyclodextrins. Pharmaceutics.

[17] Puskás I., Szente L., Szöcs L., Fenyvesi E. (2023). Recent List of Cyclodextrin-Containing Drug Products. Period. Polytech.-Chem. Eng..

